# Perceptions of Stakeholders and the Way Forward to Implementing the Family Adoption Programme in Medical Education: A Study From Assam, India

**DOI:** 10.7759/cureus.63586

**Published:** 2024-07-01

**Authors:** Anuja Baruah, Sonali G Choudhari

**Affiliations:** 1 Community Medicine, Jorhat Medical College, Jorhat, IND; 2 Community Medicine, Jawaharlal Nehru Medical College, Datta Meghe Institute of Higher Education and Research, Wardha, IND

**Keywords:** challenges, benefits, perceptions, stakeholders, competency based medical education, family adoption programme

## Abstract

Background

The Family Adoption Programme (FAP) has been introduced under the competency-based medical education curriculum wherein each medical student adopts families. The objective of this study was to determine the stakeholders' perspective and to suggest measures to make it relevant for Indian medical graduates.

Methodology

A mixed-method study was conducted among the faculty, undergraduate students, and community using prestructured, validated instruments. The quantitative data were entered into Microsoft Excel (Microsoft Corp., Redmond, WA) and analysed, while the qualitative data were coded and analysed thematically.

Results

All faculty members (12, 100%), the majority of students (49, 44.30%), and the community members strongly agreed that the policy decision to introduce the FAP was 'right'. The benefits mentioned were that FAP helps improve knowledge, psychomotor skills, attitudes and communication, attitude and behaviour skills, understanding of the social structures, health status of the community, and health-seeking behaviour of the families and provides an appropriate early clinical exposure. The challenges mentioned were selecting a site, gaining cooperation from family, communication, arrangement of logistics and transportation, getting support from teachers, difficulties in managing students in the community and coordinating among faculty, staff and students. Most faculty members recommended that the FAP should be started later in the curriculum and there should be restrictions on the number of families to be adopted. The students suggested that adequate logistics be provided as well as a reduction in the number of family visits.

Conclusion

The programme has been welcomed by most stakeholders. It requires the necessary support from the institution authorities, prior planning of visits, judicious utilization of social media, and coordination with government field-level health workers e.g. Accreditated Social Health Activists (ASHA). A comprehensive program evaluation and formulation of a standard operating module will further strengthen the programme.

## Introduction

Competency-based medical education (CBME) is designed to create an Indian medical graduate (IMG) with the necessary knowledge, skills, and attitude to assume their role as a healthcare provider to the people of India and the world [1]. The Family Adoption Programme (FAP) is an important element of CBME, wherein each medical student is required to adopt families, establish rapport, understand their living conditions, and help improve the health of the family, and in turn the community [2]. The inclusion of the FAP in the undergraduate MBBS training program aligns with the SPICE (Student-centred, Problem-based, Integrated, Community-based, Electives and Systematic) model for medical curricula [3]. In 2022, the FAP was introduced [1] and it is important to understand the stakeholders' perspectives and find ways to make the programme beneficial to all of them.

Numerous benefits of community-based medical education have been reported in the literature, such as promoting a more patient-oriented perspective that offers a broader range of learning opportunities for students to acquire knowledge, psychomotor skills, attitudes, and communication skills as well as providing students with the opportunity to learn about general and family medicine practice in a rural setting [4].

The FAP is expected to hone medical student's communication skills which are the backbone of the profession; in particular, learning to be humane and empathize with the rural population by understanding their customs and limitations as well as many positive aspects of community unity. Practical field training from the beginning will contribute to their medical education and improve their future practice. Importance may be laid on training in preventive medicine and knowledge of implementation methods of various healthcare-related schemes. Students would also be able to understand the disease profile in a rural setting that may be different from the secondary/tertiary care setting of medical colleges. In addition, they have exposure to local beliefs and faith in various alternative methods of disease management other than allopathy. This is expected to widen their vision of holistic health care and management of common ailments encountered in these settings by a family physician [5].

The present study was undertaken to study the perspective of the stakeholders of the FAP regarding the benefits and challenges of the family adoption curriculum and to find the measures to make it relevant for an IMG.

## Materials and methods

A mixed-method study was conducted at Jorhat Medical College, Assam, from September 2023 to February 2024. The qualitative component of the study was of phenomenological type, i.e., following exposure to the FAP. The participants were the faculty members (n=12) of the Department of Community Medicine, the MBBS undergraduate students of the 2021-22 batch, and 17 families and six link workers of the adopted area. Complete enumeration was used for the students and faculty participants while non-probability purposive sampling was used for the community. Among the undergraduate students, although there were a total of 125 students, only 110 student participants could be included in the study as the final sample as there were nine non-responses and six with incomplete data, amounting to a response rate of 88%.

Both quantitative and qualitative data were collected from the participants. The key informant interview (KII) format for the faculties, the focus group discussion (FGD) guide for the community members and the student survey questionnaire were prestructured and validated by the Department of Medical Education (DOME) of the study setting and by external subject experts from the Department of Community Medicine from outside Jorhat Medical College. The student survey questionnaire was converted into Google Forms and pilot-tested before data collection.

Participants having experience with FAP for at least one year were included in the study. The participants who did not give consent to participate in the study were excluded.

Approval was taken from the Institutional Ethics Committee before starting the data collection process (IEC No. - EC/ NEW/ INST/ 2020/1221). Written informed consent was obtained before conducting the KIIs with faculty members and FGDs with the community members. The initial section of the Google form containing the Survey Questionnaire stated that participation is voluntary.

Data analysis

The quantitative data and data taken using the Likert scale were entered into Microsoft Excel (Microsoft Corp., Redmond, WA), and the results were analysed and presented using tables, bar diagrams, etc. The qualitative data were coded and analysed thematically, and some of the participant perspectives were quoted.

## Results

Regarding the policy decision to introduce FAP 

**Figure 1:**
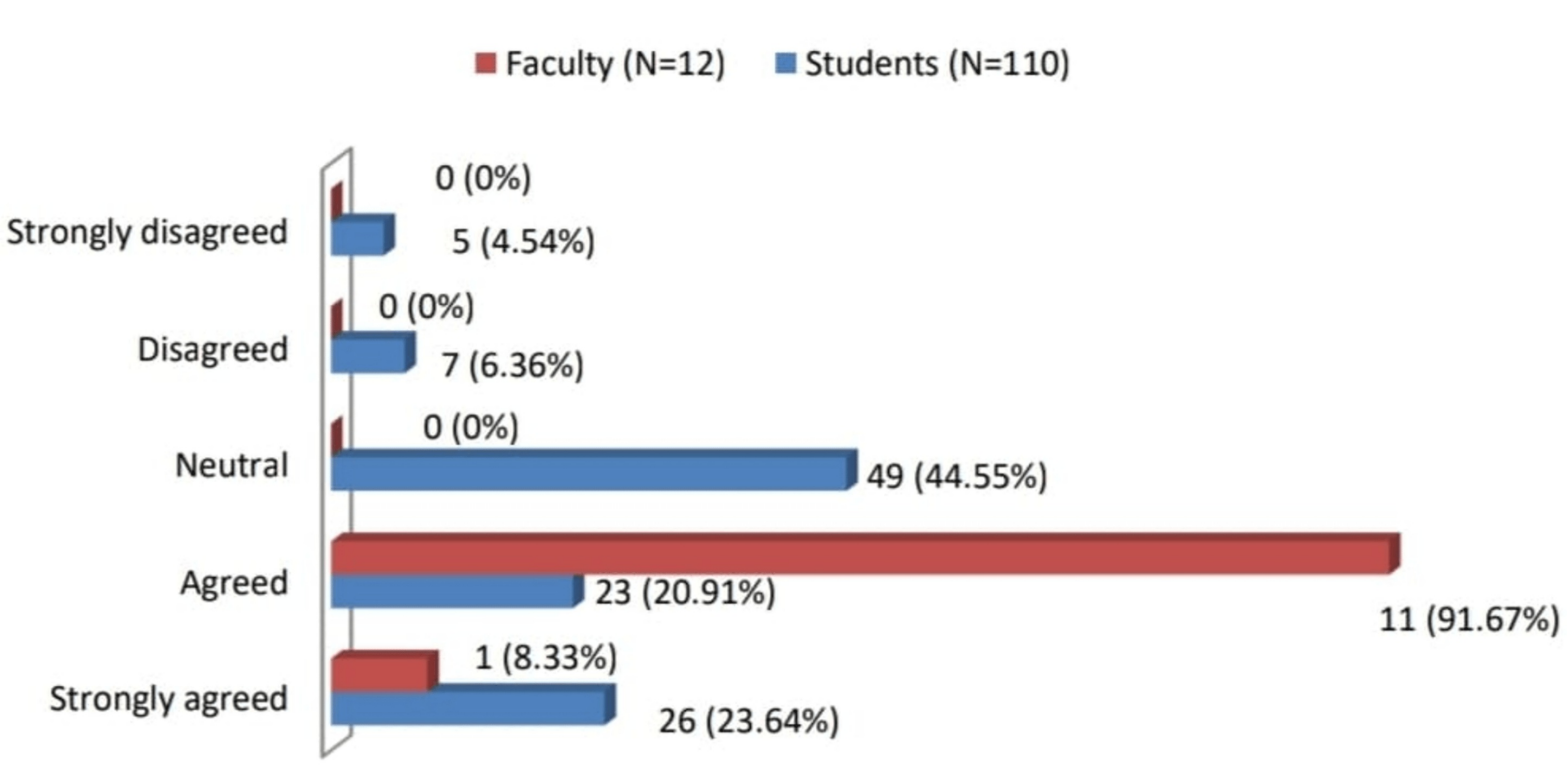
Policy decisions: FAP has rightly been introduced The data has been represented as number and percentages

Out of a total of 12 faculty participants, one faculty member strongly agreed and the rest faculty (11, 91.67%) agreed to the 'policy decision to introduce the FAP is right'. A total of 49 (44.30%) students were neutral, 26 (23.90%) strongly agreed with the decision, and another 23 (20.50%) agreed (Figure 1).

Benefits of the FAP 

**Figure 2:**
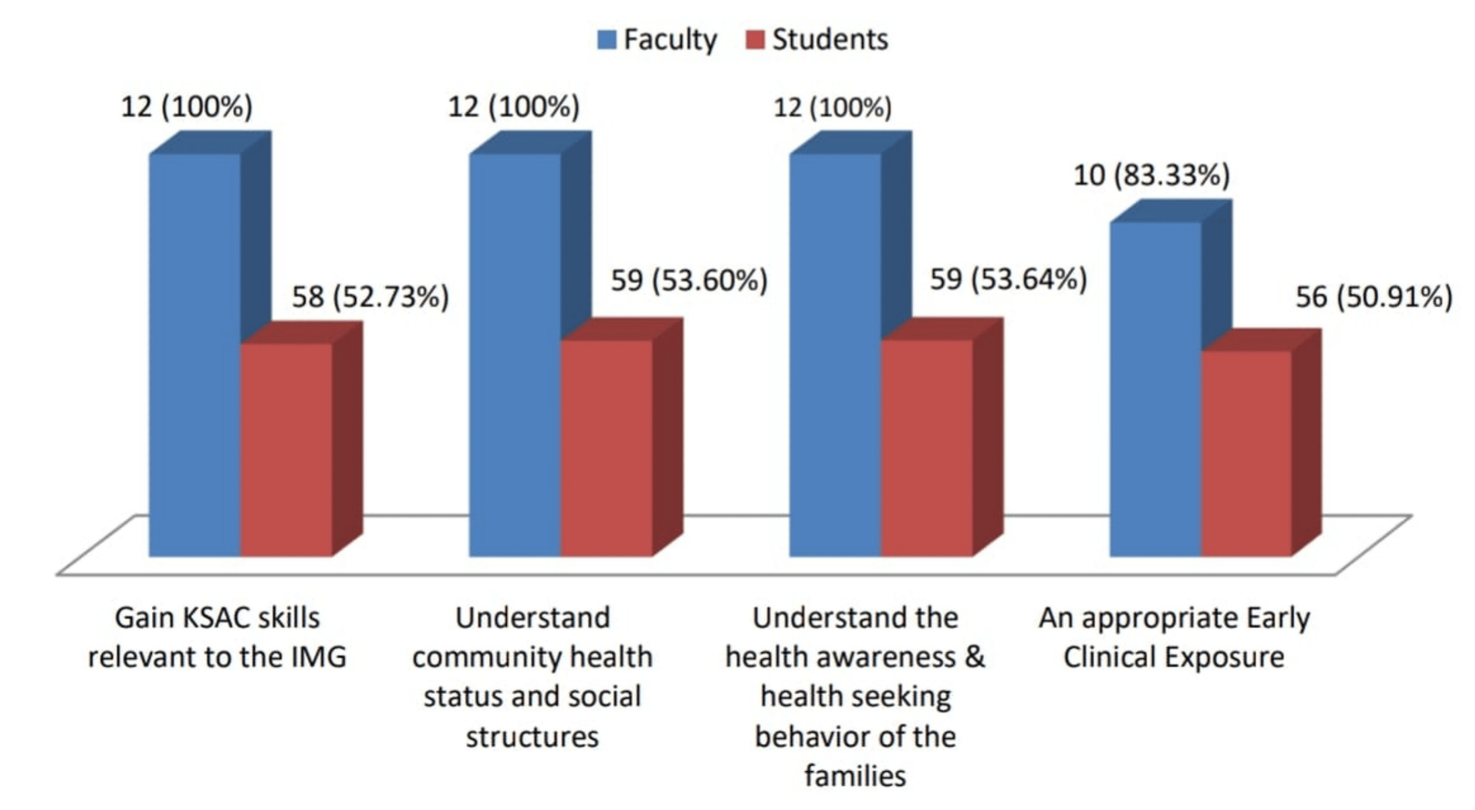
Benefits of FAP (Faculty and Students) The data has been represented as number and percentages

The majority of the participants felt that the FAP helped them gain knowledge, psychomotor skills, attitudes, and communication skills relevant to the IMG (faculty members: n=12; 100%; students: n=58; 53%), facilitates in understanding the social structures and the health status of the community (faculty members: n=12; 100%; students: n=59; 54%), promotes health seeking behaviour of the families (faculty members: n=12; 100%; students: n=59; 54%), and provides an appropriate early clinical exposure to the undergraduate medical students (faculty members: n=10;83%; students: n=56; 51%) (Figure 2).

**Figure 3:**
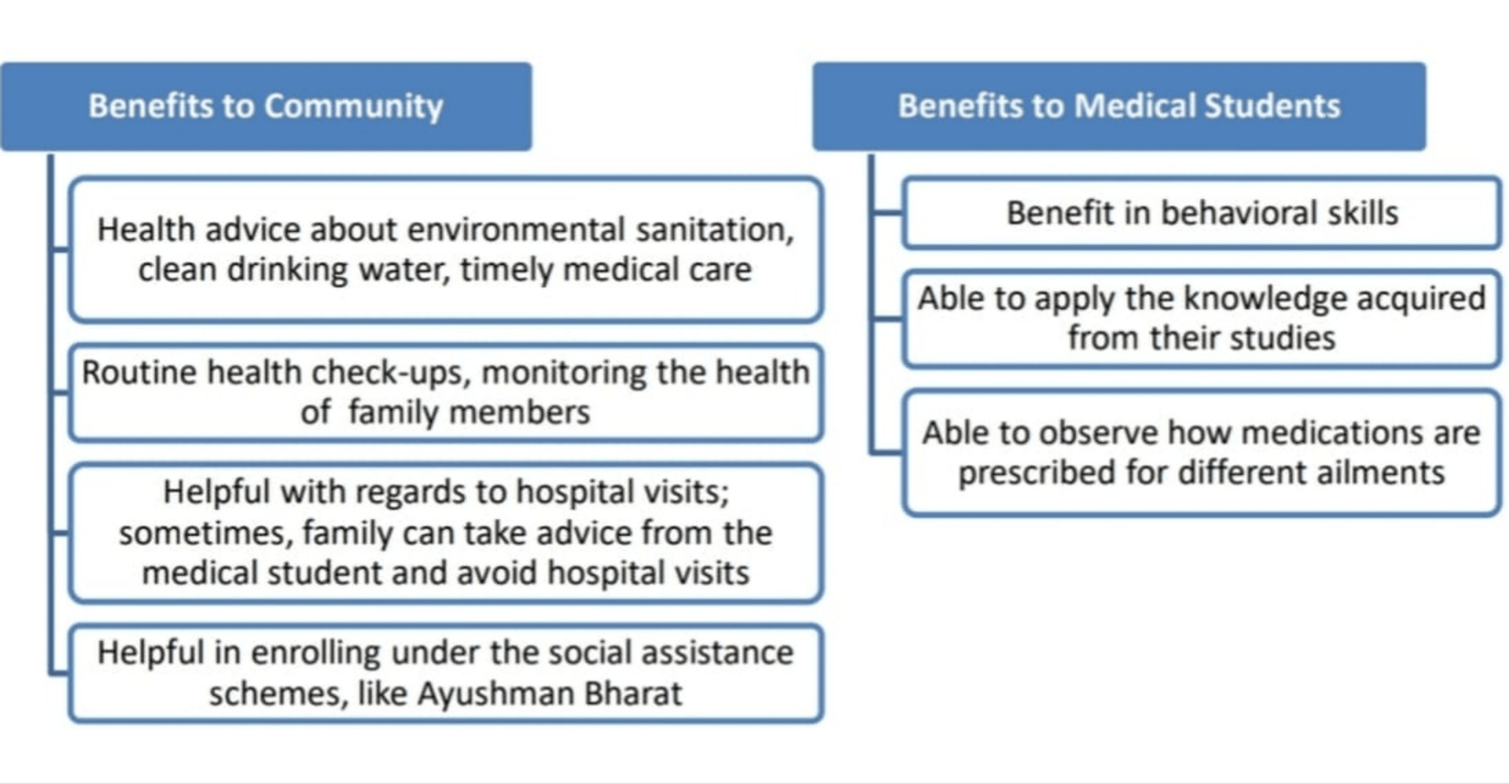
Benefits of FAP (Community FGD)

The 23 community members (which included 17 family members and six link workers) mentioned that the FAP is beneficial, as the students provide health advice, conduct routine health check-ups, help the families with hospital visits and in enrolling under central government social assistance schemes, such as Ayushman Bharat (Figure 3).

Challenges of the FAP 

The major challenges mentioned by the faculty members include the selection and finalization of the FAP site (n=11; 91.67%), difficulty in arranging for logistics and equipment for field visits (n=10; 83.33%), getting support from the community (n=10; 83.33%), difficulty to arrange for transportation of a large number of students during the family adoption visit (n=8; 66.67%), challenges in managing students in community during visits (n=6; 49.99%), difficult to have smooth coordination amongst all stakeholders faculty, staff and students (n=5; 41.67%), etc.

**Figure 4:**
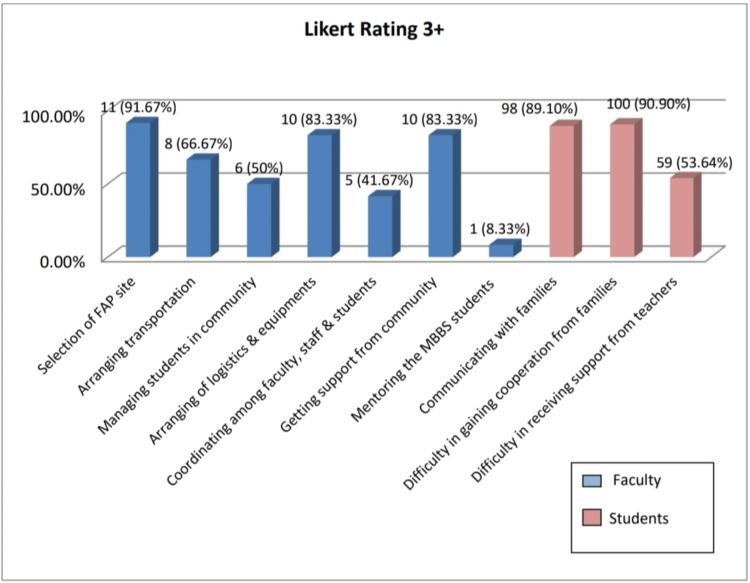
Challenges of FAP (Faculty and Students) The data has been represented as number and percentages

The key challenges as informed by students were difficulties in gaining cooperation from the family 100 (90.80%), establishing communication with the family members 99 (89.70%), and obtaining support from their teachers (n=59; 54%) (Figure 4).

**Figure 5:**
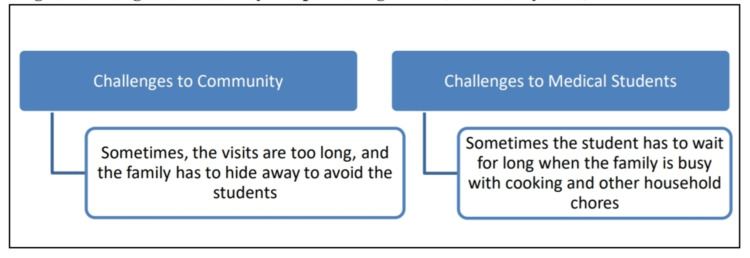
Challenges of the FAP (Community FGD)

The community members commented that sometimes the field visits get prolonged, and the student has to wait for a long in the field (Figure 5).

Recommendations regarding the FAP

Most faculties recommended that the FAP should be started later in the curriculum and there should be restrictions on the number of families to be adopted. The majority of students recommended that adequate logistics should be provided and there should be a reduction in the number of family visits (Table 1).

**Table 1:** Recommendations regarding FAP The data has been represented as numbers (n) and percentages (%). ASHA: Accredited Social Health Activist; FAP: Family Adoption Programme; SOP: standard operating procedure

Sub-themes	Faculty; n(%)	Students; n(%)
Periodic health camps	2 (16.67)	0 (0..00)
Adequate logistics	0 (0.00)	23 (20.91)
Involvement of ASHA and Anganwadi workers	1 (8.33)	0 (0.00)
Choice of families	2 (16.67)	9 (8.18)
Restriction of the number of families to be adopted	3 (25.00)	0 (0.00)
Starting the FAP later	5 (41.67)	0 (0.00)
Site selection	2 (16.67)	0 (0.00)
Active involvement of faculties	2 (16.67)	13 (11.82)
Use of local language	1 (8.33)	2 (1.82)
Timing of family visit	1 (8.33)	4 (3.64)
Scheduling of visits	1 (8.33)	13 (11.82)
Provide medical benefits	1 (8.33)	2 (1.82)
SOPs could be adopted	1 (8.33)	0 (0.00)
Reduce the number of visits	0 (0.00)	23 (20.91)
Better transportation	0 (0.00)	9 (8.18)
Awareness about family adoption	0 (0.00)	4 (3.64)
Environmental sanitation	0 (0.00)	2 (1.82)
Safety of students	0 (0.00)	4 (3.64)
Flexible program	0 (0.00)	2 (1.82)
Permanent residents	0 (0.00)	2 (1.82)
Total	12 (100)	110 (100)

Other suggestions include active involvement of faculties, proper selection of family adoption sites and choice of families, scheduling of visits, provision of better transportation facilities, and conducting periodic health camps (Table 2).

**Table 2:** Recommendations regarding FAP (Verbatim) F: faculty; S: student; numerical accompanying F or S is the serial number of the participant FAP: Family Adoption Programme; ASHA: Accredited Social Health Activist; SOP: standard operative procedure

Sub-themes	Verbatim quotes (* F= Faculty; S= Student)
Periodic health camps	“regular health camps should be conducted in FAP-adopted areas” (F1)
Adequate logistics	“logistics should be adequate” (F1); “separate fund allocation for FAP” (F3); “sufficient funds should be provided for FAP” (F10); “provide proper equipment and logistics to the students” (S9); “more equipment and more funds” (S15); “the number of instruments and equipment has to increase, eg., BP machine” (S32)
Involvement of ASHAs and Anganwadi workers	ASHA and Anganwadi workers should be involved (F1)
Choice of families	“needy families should be adopted” (F1); “adoption of only lower socioeconomic status families” (F7); “those families who live in extremely remote areas should be chosen” (S27); “choosing a location far away from any medical care, like interior villages, as those people would be highly interested owing to their distance from health care facilities” (S38); “a large rural area/ village to have some options for the students to choose a family from” (S48); “should be conducted in remote areas” (S50); “allot those families who need such type of information, some families are too educated that they already aware of their self-hygiene” (S60)
Restriction of the number of families to be adopted	“one family should be allotted to a group of 4-5 students” (F3); and “confinement of a number of families” (F7) “Instead of 3 to 5 families, it would be better if one student adopts one family, easy for everyone” (F9)
Start a little later	“delaying FAP visits to Phase 2 after clinical exposure” (F2) “starting FAP after 1 to 2 months of commencement of community medicine classes, after clinical exposure” (F5); “start after the students have the basic understanding of community medicine” (F9); “start a little later when students acquire some knowledge” (F11); “to be conducted in the second phase to avoid distraction for students” (F12)
Site selection	“site selection should be based on groundwork” (F8)
Active involvement of faculties	“involvement of faculties of other departments” (F2); “Faculties should accompany during at least first visit” (F3); “make teachers cooperate with student” (S14); “teacher companion” (S22); "teacher guidance” (S36); “teacher should introduce the student to the family” (S40); “teacher should accompany along with the student” (S53)
Use of local language	“encourage students to use the local language of the community” (F10) and “improve communication skills” (S25)
Timing of family visit	“timing of family visit should be flexible” (F8); “the timing should be such that the head of the family is present in house” (S4); “it should be conducted at a time when the family members are free” (S24); “it should not be in the morning hours” (S32)
Scheduling of visits	“schedule visits in a continuous way” (F12); “increasing the number of visits” (S3); “students should visit the adopted families regularly” (S16); “more interaction with the family” (S20); “continuous communication with the family by the students” (S59)
Provide medical benefits	“medical college can provide a card for free treatment for one year for the allotted family” (F3); “provide benefits like medicines and consultation” (S5)
SOPs can be adopted	"Standard operative procedure can be adopted" (F6)
Reduce the number of visits	“less number of visits” (S8)
Awareness about family adoption	“society should feel the importance of family adoption to improve the health care status of society” (S44); “Students should take this programme with discipline and utmost seriousness” (S45) “making people aware of the importance of FAP improving public health” (S45)
Environmental sanitation	“proper waste management and improve water supply” (S17)
Safety of students	“safety of the students” (S67)
Flexible program	“to keep the program flexible as different families have different issues which cannot be solved by particular set guidelines” (S41)
Permanent residents	“to make sure how many families are originally from the village and how many are living in rent because those living in rented houses sometimes are not available during the next visit and create a lot of problems” (S61)

The community members suggested that the timings of the family visits should be convenient for the family. They suggested that the college authority can help by coordinating with the government in the provision of clean drinking water to households and seasonal DDT spraying against mosquitoes. They should also organize health camps periodically. The community health workers suggested that health advice on nutritious food and healthy weights, intake of iron-folic acid supplementation among pregnant women, family planning, environmental cleanliness, and personal hygiene should be incorporated, and expressed that especially if the students advise the adolescents on avoidance of substance abuse and addictive use of mobile phones, it probably will be better listened to because they are of the same age group.

## Discussion

This study aims to study the perspective of the stakeholders of the FAP regarding the benefits and challenges of the family adoption curriculum and to find the measures to make it relevant for an Indian medical graduate

According to Shrivastava et al. [4], medical education aims to train students in such a way that they are empowered to meet the health needs of the population. All the faculty members, the majority of undergraduate students, and the community were found to welcome the policy decision about the introduction of the FAP. In a similar study by Yalamanchili et al. [6], the participants including the faculty, undergraduate and postgraduate students, and field workers suggested that the reforms should be gradual and sustained, and there should be some flexibility to maintain the quality.

All the participants mentioned that the FAP has many benefits. The faculty members and the students commented that it helps to acquire the knowledge, skills, attitudes, and communication skills relevant to Indian medical graduates, and to understand the health needs of the community as well as their health-seeking behaviours. It provides a very enriching early clinical exposure to the MBBS undergraduate students. It was seen that the community members also found the family adoption visits beneficial. They commented that they benefit from health advice provided by the students. The students also conduct routine health check-ups, help families with hospital visits and assist them in enrolling in social assistance schemes. Similar findings have been reported by other studies. In the study by Yalamanchili et al. [6], the participants, namely, the faculty members, the undergraduate and postgraduate students, and the field workers commented that the students learnt about patients’ backgrounds and significant health problems and encountered several misconceptions about health among the general population. In the study by Ganapathy [7], the participants loved the hands-on experience, and the experience of being able to ‘be a doctor’ and it was a good clinical as well as primary health care exposure. The community members benefitted from the health advice and commented that the students communicated well with the people, and some of the health problems were solved at the doorsteps. Family adoption is instrumental in the improvement of self-perceived communication skills and the ability to help the community with their health problems [8].

The faculty participants in the present study indicated that 'the selection of a family adoption site' was the most important challenge, while the student participants stated that communication with the family members and difficulty in gaining cooperation from the family was the most important challenge. Other challenges mentioned by the participants include an arrangement of logistics and transport facilities, and of course, difficulty in coordinating between the faculty, staff and students and in getting inter-stakeholders’ support. Regarding community feedback, it was found that the community members felt that sometimes the visits were too long, or at other times, the student had to wait for a long time.

The challenges mentioned by the participants resonated with the findings in other studies. In the study by Yalamanchili et al. [6], the participants indicated a shortage of logistics and transport facilities, human resources, and noted that the reduced faculty was already burdened by self-directed learning, small group discussions, and by any form of harm or bad influence on the students by the families. In the study by Ganapathy [7], the challenges encountered were the new learning environment, personal challenges, language barrier, having to work independently, insufficient background medical knowledge, group dynamics, limited resources, filling log book and limited time. Further, the lack of sensitisation of stakeholders and inadequate planning were identified as the predominant challenges in the implementation of CBME [8].

Most faculties recommended that the family adoption program should be started a bit later in the curriculum. Though a few faculty members stated about “starting FAP after one to two months of commencement of community medicine classes, after clinical exposure” (F5), to “start a little later when students acquire some knowledge” (F11) and “start after the students have the basic understanding of community medicine” (F9), another two faculties commented about “delaying FAP visits to Phase 2 after clinical exposure” (F2) and “to be conducted in Phase 2 to avoid distraction for students” (F12).

The faculty members also stated that there should be a restriction on the number of families to be adopted, as one of them said “Instead of three to five families, it would be better if one student adopts one family, easy for everyone” (F9).

The majority of students recommended that adequate logistics should be provided and there should be a reduction in the number of family visits. The students were found to be concerned about logistics, as per the following statements: “provide proper equipment and logistics to the students” (S9), “the number of instruments and equipment has to increase, eg., BP machine” (S32), “more equipment and more funds” (S15), etc. In a study by Amogha et al. [9], one of the students quoted the reason for noncooperation as “according to the locals, after collection of information by the students, no action was taken by the concerned authorities to curb the health issues of the families such as free medication or consultation” while other student said, “Language barrier and trust”.

Regarding active involvement of faculties, the participants were found to speak as “teacher should introduce the student to the family” (S40), “faculties should accompany during at least first visit” (F3), “make teachers cooperate with student” (S14), “teacher companion” (S22) and also about “involvement of faculties of other departments” (F2). 

Valuable comments were provided by the participants regarding the selection of family adoption sites and the choice of families. the participants suggested that “needy families should be adopted” (F1), “adoption of only lower socioeconomic status families” (F7), “those families who live in extremely remote areas should be chosen” (S27), “a large rural area/ village to have some options for the students to choose a family from” (S48). Some participants were found to be even more precise in commenting about “choosing a location far away from any medical care, like interior villages, as those people would be highly interested owing to their distance from health care facilities” (S38) and to “allot those families who need such type of information, some families are too educated that they are already aware of their self-hygiene” (S60). The participants commented on the proper scheduling of visits, provision of better transportation facilities and conducting periodic health camps.

The participants also felt the need for awareness amongst the students and the community regarding the family adoption programme. They commented that “society should feel the importance of family adoption to improve the health care status of society” (S44), about “making people aware of the importance of FAP for improving public health” (S45) and that “students should take this programme with discipline and utmost seriousness” (S45). They also suggested taking assistance from the ASHAs & Anganwadi Workers. Another valuable suggestion was about the use of the local language. They felt the need to“encourage students to use the local language of the community” (F10) and to “improve communication skills” (S25).

The community members suggested that the timings of the family visits should be at the convenience of the family. This was agreed upon by some faculty and student participants as the “timing of family visit should be flexible” (F8), “it should be conducted at a time when the family members are free” (S24), “the timing should be such that the head of the family is present in house” (S4) and that “it should not be in the morning hours” (S32).

The community members further suggested that the college authority can help by coordinating with the government in the provision of certain facilities such as clean drinking water to households and seasonal DDT spraying against mosquitoes. They should also organize health camps periodically. The community health workers suggested that health advice on nutritious food and healthy weights, intake of iron-folic acid supplementation among pregnant women, family planning, environmental cleanliness and personal hygiene should be incorporated, and expressed a preference for students to advise adolescents in their community regarding avoidance of substance abuse and the addictive use of mobile phones, as they felt that the relative closeness in age would make teenagers more receptive to counselling regarding these matters. In the study by Yalamanchili et al. [6], the participants suggested measures such as bringing management on board for logistics, as well as thorough planning and orientation for one to two days to make FAP more effective. It was suggested the undergraduate students should clarify they’re first‑year students, not doctors, and that there should be tertiary care follow‑up in the hospital and active health interventions like alcohol de-addiction.

Limitations and recommendations

The findings of the study are significant pilot findings on the experience of the various stakeholders, namely, the Faculty of Community Medicine, the undergraduate MBBS students and the community members regarding the FAP. The study is insufficient to enlighten on the product of the family adoption curriculum, in its contribution to the attainment of the competencies of the Indian medical graduate but provides much evidence about the experience and acceptance of the programme, and can provide important suggestions towards it. One very thought-provoking issue brought forward by the study is about the timing of the introduction of the programme in the undergraduate curriculum as the majority of participants were found to comment that it should be introduced later in the Phase 1 year. Further research needs to be conducted towards the development of student assessment tools, the development of a structured module for the family adoption programme and a comprehensive program evaluation.

## Conclusions

The inclusion of the FAP in the undergraduate MBBS training program aligns with the SPICE (Student-centred, Problem-based, Integrated, Community-based, Electives and Systematic) model for medical curricula. Community-based education for health professionals is effective in fostering health personnel who are responsive to community needs. It is also effective in reducing hospital referrals. and improving communication and clinical management skills. The programme has been welcomed by most stakeholders and has been found to help improve communication and rapport-building skills, comprehend problems at the community level and health-seeking behaviour, provide early clinical exposure, and is also beneficial to the community. For the smooth implementation of the FAP, the college authorities, concerned faculty and other stakeholders have to be committed to providing the necessary support, the visits should be planned in advance, and there should be judicious use of social media and utilization of help from government field level health workers such as ASHAs. A comprehensive program evaluation and formulation of a standard operating module will strengthen the family adoption component of the CBME curriculum.
